# MicroRNA expression profiling in Imatinib-resistant Chronic Myeloid Leukemia patients without clinically significant ABL1-mutations

**DOI:** 10.1186/1476-4598-8-69

**Published:** 2009-09-01

**Authors:** Edurne San José-Enériz, José Román-Gómez, Antonio Jiménez-Velasco, Leire Garate, Vanesa Martin, Lucia Cordeu, Amaia Vilas-Zornoza, Paula Rodríguez-Otero, María José Calasanz, Felipe Prósper, Xabier Agirre

**Affiliations:** 1 Foundation for Applied Medical Research, Division of Cancer, Area of Cell Therapy and Hematology Service, Clínica Universitaria, Universidad de Navarra. Spain; 2 Department of Hematology. Hospital Reina Sofía e Instituto Maimónides de Investigación Biomédica, Córdoba. Spain; 3 Department of Hematology, Hospital Carlos Haya, Málaga, Spain; 4 Department of Genetics, University of Navarra, Spain

## Abstract

The development of Imatinib Mesylate (IM), the first specific inhibitor of BCR-ABL1, has had a major impact in patients with Chronic Myeloid Leukemia (CML), establishing IM as the standard therapy for CML. Despite the clinical success obtained with the use of IM, primary resistance to IM and molecular evidence of persistent disease has been observed in 20-25% of IM treated patients. The existence of second generation TK inhibitors, which are effective in patients with IM resistance, makes identification of predictors of resistance to IM an important goal in CML. In this study, we have identified a group of 19 miRNAs that may predict clinical resistance to IM in patients with newly diagnosed CML.

## Introduction

The development of Imatinib Mesylate (IM), the first specific inhibitor of BCR-ABL1, has had a major impact in patients with Chronic Myeloid Leukemia (CML) [1]. Treatment with IM induces a complete hematological and cytogenetic response in more than 90 and 80% of newly diagnosed patients with chronic phase CML respectively, [2] which has established IM as the standard therapy for CML. Despite the clinical success obtained with the use of IM, primary resistance to IM and molecular evidence of persistent disease has been observed in 20-25% of IM treated patients [1]. Different mechanisms of resistance have been described, including the presence of point mutations in the tyrosine kinase domain of *BCR-ABL1*, amplification and overexpression of *BCR-ABL1*, overexpression of efflux transporters (such as *ABCB1*, also known as MDR1 or p-glycoprotein, and *ABCG2*) and underexpression of uptake transporters (such as *SLC22A1*, also known as hOCT1) [1]. However, it is currently impossible to predict whether a patient will develop resistance to IM. The existence of second generation TK inhibitors, which are effective in patients with IM resistance, makes identification of predictors of resistance to IM an important goal in CML.

MicroRNAs (miRNAs) are non-coding, single-stranded RNAs of 21-25 nucleotides that have recently been implicated in the regulation of a number of biological processes such as development, differentiation, apoptosis, proliferation and hematopoiesis. They regulate gene expression by promoting degradation of the mRNA or repressing its translation [3]. In addition, miRNAs have been implicated in the development of human cancers, either as tumor suppressors or as oncogenes. Aberrant miRNA expression has recently been described for a variety of solid tumors (lung, breast or colorectal cancer among others) and hematological malignancies (chronic lymphocytic leukemia, B-cell lymphomas, acute promyelocytic leukemias, acute lymphocytic leukemia and CML). In CML, abnormal expression of several miRNAs has been described (*miR-15a*, *miR-16*, *miR-142*, *miR-155*, *miR-181*, *miR-221*, *let7a *and the polycistronic *miR-17-92 *cluster) [4-6] and we have currently demonstrated that down-regulation of *miR-10a *leads to an abnormal proliferation of CML cells through regulation of *USF2 *gene expression [3]. A role for microRNA expression as a prognostic factor in various tumors has only been recently described, [7] while no information is available regarding their involvement in the response to chemotherapy. Recent studies suggest that *miR-15b *and *miR-16*, and *miR-27a *and *miR-451 *modulate multidrug resistance by targeting *BCL2 *and *MDR1 *respectively [8,9].

## Findings

With the objective of deciphering a potential miRNA expression signature associated with IM resistance, we analyzed the expression profile of 250 miRNAs by Q-RT-PCR using TaqMan methodology (PE Applied Biosystems, Foster City, CA) using bone marrow mononuclear cells from patients with Ph^+ ^CML at diagnoses (n = 8). All patients were treated with identical doses of IM (400 mg daily) as first-line therapy. Three patients were considered to be primary resistant (less than partial cytogenetic response (Ph+ > 35%) after 12 months on IM; 400 mg/day for the first six months and 800 mg/day thereafter) while the other 5 patients showed a complete cytogenetic response at 12 months. In every case, we examined the sample at diagnosis and twelve months after treatment with IM for the presence of the clinically significance mutations T315I, Y253H, Y253F, E225K and E255V using allele-specific oligonucleotide polymerase chain reaction (ASO-PCR), and none of these mutations was detected (data not shown). All studies were approved by the Research Review Boards at the University of Navarra. Informed consent was obtained from all the patients.

RNA extraction of nucleated cells and reverse transcription were carried out as described [3]. Briefly, total RNA was extracted with Ultraspec (Biotecx, Houston, TX, USA) following the manufacturer's instructions. 5 ng of total RNA was used to synthesize a specific cDNA of each analyzed miRNA using stem-loop miRNA-specific RT primer, according to the TaqMan MicroRNA Assay protocol (Applied Biosystems, Foster City, CA). Expression of 250 miRNAs was analyzed using specific primers and TaqMan probe for each miRNA according to the TaqMan MicroRNA Assay Protocol (Applied Biosystems, Foster City, CA). Q-RT-PCR was performed using an Applied Biosystems 7300 Sequence Detection system as described [3]. Expression of miRNA was normalized using the expression of the housekeeping gene *RNU6B*. Expression of miRNAs in every patient is included in the Additional file 1. Relative quantification of expression of analyzed miRNAs was calculated with the 2-^ΔΔCt ^method (Applied Biosystems. User Bulletin N°2 (P/N 4303859)). Data are presented as log 2-^ΔΔCt ^of the relative quantity of miRNAs, normalized and compared with the expression in samples obtained from patients that responded to IM. A supervised analysis using the SAM algorithm (Significant Analysis of Microarrays) was performed in order to identify miRNAs with statistically significant changes in expression between both groups.

**Figure 1:**
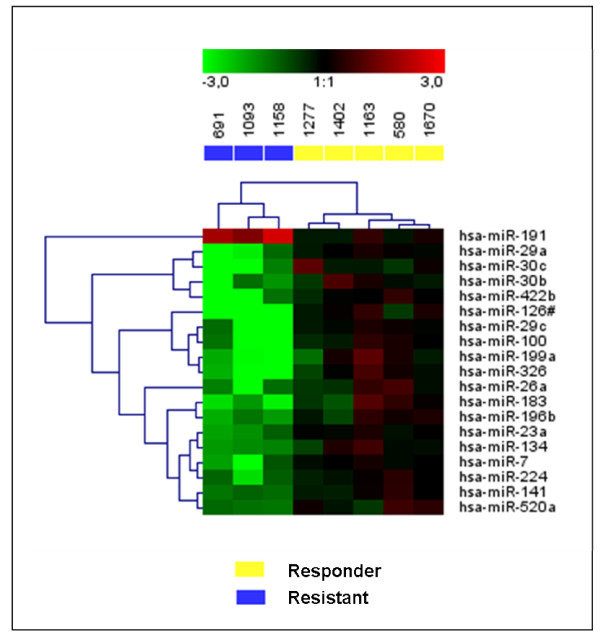
**Hierarchical cluster analysis of miRNA expression in resistant and responder CML patients**. Cluster analysis of expression of the 19 miRNAs differentially expressed between resistant and responder CML patients using Genesis software. The dendrogram is shown at the top of the figure.

Using both methods, relative quantification and supervised analysis, we identified 19 miRNAs differentially expressed between resistant and responder samples: 18 of them were down-regulated (*hsa-miR-7*, *hsa-miR-23a*, *hsa-miR-26a*, *hsa-miR-29a*, *hsa-miR-29c*, *hsa-miR-30b*, *hsa-miR-30c*, *hsa-miR-100*, *hsa-miR-126*#, *hsa-miR-134*, *hsa-miR-141*, *hsa-miR-183*, *hsa-miR-196b*, *hsa-miR-199a*, *hsa-miR-224*, *hsa-miR-326*, *hsa-miR-422b *and *hsa-miR-520a*) and only one was up-regulated (*hsa-miR-191*) in resistant CML patients. To demonstrate whether this set of miRNAs may classify properly both groups, we performed an unsupervised cluster analysis using Genesis software using the results obtained with the 8 patients included in the analysis. Hierarchical clustering based on the average-linkage method with the centered correlation metric was used. The cluster unsupervised analysis based on differentially expressed miRNAs generated a tree that clearly separates both groups (Figure 1) suggesting that this miRNA profile could distinguish the IM-response in CML patients.

In order to determine potential genes and signaling pathways implicated in resistance to IM, we analyzed predicted targets of the 19 miRNAs differentially expressed using four different algorithms (mirBase, miRanda, TargetScan and PicTar). Because each of the four approaches generated an unpredictable number of false positives, results were intersected to identify the genes commonly predicted by at least three of the algorithms. Among all the possible targets for these miRNAs we found several membrane transporters that belong to the ATP binding cassette (ABC) superfamily of transmembrane transporters, which have been implicated in resistance to chemotherapy. These transporters are ABCC5, ABCA1 and ABCB6, potential targets of *hsa-miR-199a*, *hsa-miR-183 *and *hsa-miR-29c *respectively. Our own unpublished studies indicate that up-regulation of *ABCA1 *is observed in CML patients that develop resistance to IM (data not shown), suggesting that alterations of ABC transporters mediated by miRNAs might be a mechanism of resistance to IM. Other targets of the differentially expressed miRNAs such as RAB11A (possible target of *hsa-miR-520a*) or SIRT1 (possible target of *hsa-miR-199a*) have recently been implicated in resistance to IM [10,11].

In conclusion, we identify a group of 19 miRNAs that may predict clinical resistance to IM in patients with newly diagnosed CML. Further studies are nevertheless required to validate the role of these miRNAs as predictors for resistance to IM and to identify miRNAs targets and their function in resistance to IM in CML.

## Competing interests

The authors declare that they have no competing interests.

## Authors' contributions

Conception and design: ESJE, JRG, FP, XA. Provision of study materials or patients: JRG, AJV, VM, PRO, MJC, FP. Collection and assembly of data: ESJE, JRG, AJV, LG, VM, LC, AVZ, PRO, MJC, FP, XA. Data analysis and interpretation: ESJE, JRG, LG, LC, AVZ, FP, XA. Manuscript writing: ESJE, FP, XA. Final approval of manuscript: ESJE, JRG, AJV, LG, VM, LC, AVZ, PRO, MJC, FP, XA.

## Supplementary Material

Additional file 1 **Expression of 250 miRNAs in resistant and responder CML patients**. The data provided represent the expression of microRNA. Click here for file
