# Supplementary material for: MicroRNA expression profiling in Imatinib-resistant Chronic Myeloid Leukemia patients without clinically significant ABL1-mutations
Source: Mol Cancer. 2009 Sep 1;8:69. doi: 10.1186/1476-4598-8-69 (PMC2743636; doi:10.1186/1476-4598-8-69)
Supplement: Additional file 1 — Expression of 250 miRNAs in resistant and responder CML patients. The data provided represent the expression of microRNA. [file 1476-4598-8-69-S1.pdf]

TABLE S1: Expression of 250 miRNAs in resistant and responder CML patients. ΔcT: cT of miRNA-cT of RNU6B. (RNU6B was used as internal calibrator).

	RESISTANTS			RESPONDERS				
**AB Assay Name**	**ΔcT 1158**	**ΔcT 1093**	**ΔcT 691**	**ΔcT 1670**	**ΔcT 1277**	**ΔcT 1402**	**ΔcT 1163**	**ΔcT 580**
let7a	-3.6578	-7.7402	-3.6749	-7.87	-6.1142	-5.9813	-6.9808	-7.6708
let7b	-3.2565	-7.2323	-3.3563	-7.41	-4.4802	-5.6723	-6.8625	-6.3621
let7c	1.8229	-0.7049	4.2308	-2.65	0.9093	-0.3241	-2.2893	-0.8646
let7d	-0.0444	11.9978	15.404	-2.47	-1.7206	-2.5362	-4.2572	-3.8144
let7e	7.7853	-22.7194	-3.2469	2.72	5.6746	2.8104	0.6104	2.2375
let7f	-1.4479	-5.3128	-1.519	-5.15	-2.6048	-4.5991	-5.7045	-5.1793
let7g	-3.2257	-4.6804	-20.9406	-6.48	-4.7666	-5.9481	-6.9448	-14.9345
let7i	-0.2315	-1.4066	0.4985	-4.14	-3.3187	-1.6379	-4.7998	-4.5638
hsa-miR-1	8.7509	5.8948	-11.9781	2.5	6.5736	5.2558	0.0083	1.5192
hsa-miR-7	4.7084	11.9978	7.6511	1.29	1.9794	1.2451	0.4672	1.8119
hsa-miR-9	4.6289	1.0629	4.6137	1.06	3.3572	2.6003	3.4736	4.0769
hsa-miR-10a	5.3556	0.8265	6.1015	-0.44	4.6122	3.3429	-1.468	2.1587
hsa-miR-10b	5.3086	3.1462	5.0814	2.52	5.6475	4.776	0.8311	1.4705
hsa-miR-15a	0.1398	-3.8572	-0.5219	-3.92	-2.6091	-3.165	-4.1001	-4.3607
hsa-miR-15b	-4.1848	-10.1508	-8.1988	-8.14	-5.6936	-10.8552	-9.6498	-12.5315
hsa-miR-16	-7.2651	-10.485	-4.1009	-10.8	-9.7097	-12.2681	-10.9989	-22.9731
hsa-miR-17-3p	3.3476	2.2757	4.893	0.67	0.4326	-0.2443	-0.8652	-1.0573
hsa-miR-17-5p	-2.1721	-3.5384	1.0997	-5.21	-4.4021	-4.4424	-8.7181	-5.9787
hsa-miR-18a	-1.0606	-2.6471	-10.5612	-3.48	-2.2014	-11.9732	-4.6244	-3.1732
hsa-miR-19a	-2.4581	-6.535	-2.1114	-6.22	-4.9858	-5.7065	-8.9738	-6.2386
hsa-miR-19b	-6.2381	-8.3931	-8.4577	-7.66	-6.8165	-7.0912	-9.6546	-8.2557
hsa-miR-20a	-4.369	-11.1765	-7.4202	-10.31	-6.7776	-6.8852	-10.8173	-12.8582
hsa-miR-20b	-0.2684	-3.2689	0.5759	-3.34	-2.0813	-2.2185	-4.672	-3.9987
hsa-miR-21	-2.8961	-5.7757	-2.1651	-5.53	-4.4063	-9.8649	-9.5867	-5.7347
hsa-miR-22	1.0548	-11.6977	0.8117	-0.41	-0.2423	-1.707	-3.8277	-2.9608
hsa-miR-23a	0.9352	2.9154	3.5909	-2.99	-2.4956	-2.4686	-3.8689	-2.1111
hsa-miR-23b	3.5524	-0.7996	1.4948	-0.43	-0.2646	-1.3497	-3.2186	-13.789
hsa-miR-24	-7.3552	-8.2663	-3.4095	-7.3	-5.9902	-8.8954	-9.1225	-7.4319
hsa-miR-25	-4.3224	-6.9156	-4.1449	-5.54	-5.2021	-11.483	-4.7763	-11.8049
hsa-miR-26a	-5.2111	11.9978	-4.5276	-8.99	-7.3037	-7.6217	-11.5725	-12.2237
hsa-miR-26b	-13.1355	-6.1387	-3.347	-8.48	-9.3545	-12.2785	-10.4723	-8.0727
hsa-miR-27a	-9.5095	-21.1697	15.404	-6.97	-6.3371	-9.8191	-7.2188	-7.8591
hsa-miR-27b	1.5121	-3.6372	4.105	-1.78	-0.9787	-1.8026	-3.6076	-3.8347
hsa-miR-28	1.6331	-1.959	1.5902	-1.48	-0.3457	-12.206	-1.4358	-1.408
hsa-miR-29a	-1.0203	4.2363	15.404	-4.74	-4.1372	-5.2041	-6.1307	-5.4215
hsa-miR-29b	1.3263	-2.0648	1.1362	-0.29	-0.9343	0.2168	-14.5667	-0.9928
hsa-miR-29c	12.7493	6.4795	-0.0389	-4.03	-3.316	-3.9003	-5.3865	-4.8419
hsa-miR-30a-3p	2.9007	-0.1417	2.6049	-0.44	0.6691	1.1556	0.3214	0.096
hsa-miR-30a-5p	-10.6334	-11.8165	-3.588	-5.47	-4.5402	-10.7634	-8.6234	-11.9483
hsa-miR-30b	-2.4478	-3.8857	7.6434	-7.2	-6.0259	-11.2833	-9.2976	-7.6396
hsa-miR-30c	-1.0476	8.1969	15.404	-6.65	-9.6782	-5.0602	-5.1074	-4.0084
hsa-miR-30d	-1.8755	-6.0871	-12.2794	-4.35	-3.1717	-11.0978	-5.6684	-4.995
hsa-miR-30e-3p	-1.9607	-0.6131	-0.9197	-3.88	-3.3061	-2.7647	-19.2259	-4.5063
hsa-miR-30e-5p	0.2651	-4.2337	-1.7559	-2.23	-0.7165	-1.4103	-3.6624	-5.2425
hsa-miR-31	6.5579	1.594	7.1756	0.47	3.6999	1.5644	0.9581	-0.7975
hsa-miR-32	0.6752	-1.9887	-10.8365	-3.28	-0.9854	-1.747	-3.4928	-3.3293
hsa-miR-33	8.636	7.5294	13.8515	6.18	5.6179	7.6861	2.4842	3.3746
hsa-miR-34a	3.8374	-14.802	7.6061	2.35	2.7705	4.6266	0.3283	2.3161
hsa-miR-34b	8.2165	4.2353	7.3552	5.53	6.2321	4.9675	7.7033	7.1317
hsa-miR-34c	7.6169	5.4579	7.8899	9.69	6.1496	4.4709	5.6219	7.0524

hsa-miR-92	-16.834	11.9978	6.7762	-8.52	-6.4035	-9.4039	-9.387	-7.3954
hsa-miR-93	-3.9024	-5.3879	-3.6654	-8.52	-5.4147	-5.9387	-7.0716	-7.1891
hsa-miR-94	8.8759	5.0684	8.268	4.78	7.277	3.2617	4.4448	2.9905
hsa-miR-96	13.9407	4.1449	9.45	5.12	7.1011	8.2073	2.5898	2.3961
hsa-miR-98	3.0882	-0.6559	3.4046	-1.7	0.3474	-1.2854	-3.1415	-2.3236
hsa-miR-99a	2.3958	0.1911	3.8738	0.68	1.7458	0.9814	-1.504	-0.1764
hsa-miR-99b	2.6029	-1.2527	3.8546	1.17	3.0874	-0.2159	-2.7808	-0.8415
hsa-miR-100	14.5346	11.9978	4.7073	0.19	1.904	1.2985	-1.5874	-0.7794
hsa-miR-101	-11.0344	-3.5965	1.5899	-3.42	-3.0385	-2.0649	-4.9265	-4.1122
hsa-miR-103	-2.7835	-5.6051	-3.4123	-5.29	-4.4099	-5.4138	-6.4978	-5.8165
hsa-miR-106a	-9.7473	-5.4004	-6.9683	-6.27	-4.3909	-3.9158	-8.6291	-5.7053
hsa-miR-106b	-3.3032	-11.1353	-4.3053	-6.34	-4.38	-4.6942	-6.5962	-6.8538
hsa-miR-107	4.2841	-0.3051	1.5456	0.23	0.9656	0.4813	-1.8774	-0.9911
hsa-miR-122a	14.5346	11.9978	15.404	11.41	9.3648	12.956	10.9907	12.5407
hsa-miR-124a	14.5346	11.9978	9.9555	11.41	10.6235	12.956	12.3404	9.1946
hsa-miR-125a	-1.4478	-5.9099	0.1783	-3.47	-1.0081	-3.7436	-9.0308	-3.9995
hsa-miR-125b	1.5802	-0.9353	2.4517	-0.93	0.7888	-0.4966	-2.4937	-1.1606
hsa-miR-126#	14.5346	9.2603	14.37	-3.53	-1.4415	-2.752	-4.3252	-0.1514
hsa-miR-127	7.7275	4.1737	5.4111	2.94	3.9533	0.3972	0.6047	3.1183
hsa-miR-128a	8.0966	6.2006	8.4222	4.26	6.4758	5.1438	3.0887	4.0964
hsa-miR-128b	14.5346	11.9978	9.6101	6.18	6.0178	7.956	6.7689	6.4918
hsa-miR-129	12.8981	11.0233	15.404	11.41	13.4642	12.956	5.2214	10.4794
hsa-miR-130a	1.8704	-0.5836	3.0791	-2.85	-1.2921	-2.0075	-5.1698	-2.8242
hsa-miR-130b	1.7826	-1.8974	2.2034	-1.2	-0.8939	-1.9496	-4.0032	-2.6064
hsa-miR-132	4.7647	1.8051	6.5074	-0.41	1.3164	0.2863	-0.5852	-0.2947
hsa-miR-133a	8.0143	2.8826	7.5327	3.83	6.445	4.6264	0.4909	1.4865
hsa-miR-133b	11.5346	-1.0651	11.404	1.43	2.8754	3.2636	-0.8627	-0.1271
hsa-miR-134	9.4357	9.7279	10.3037	4.83	6.8821	3.0083	1.8297	4.7141
hsa-miR-135a	5.6385	3.644	7.8707	5.14	3.3382	3.182	-0.457	3.2583
hsa-miR-135b	-12.0978	7.0406	13.0784	5.87	6.2877	4.8971	3.0029	4.8995
hsa-miR-137	14.5346	11.9978	15.404	11.41	11.7235	10.9024	8.3404	7.1798
hsa-miR-138	11.7823	10.2128	9.1732	7.41	9.4642	6.2972	10.8974	8.5407
hsa-miR-139	7.6756	7.9978	6.4226	4.07	5.8087	2.1182	0.4398	3.3013
hsa-miR-140	-2.4182	-5.8385	-2.4535	-6.28	-4.2538	-4.6114	-5.064	-4.9663
hsa-miR-141	7.0329	6.6861	7.4162	2.66	3.9151	4.0083	2.2764	1.0371
hsa-miR-142-3p	-8.4557	-9.4484	-8.2303	-11.46	-10.311	-12.4164	-11.1714	-15.4334
hsa-miR-142-5p	-4.9546	-7.7587	-6.0406	-7.79	-10.2418	-19.082	-14.5426	-8.1026
hsa-miR-143	-0.275	-2.1537	-0.4621	-1.12	-0.9297	-1.0287	0.3648	-1.8893
hsa-miR-145	-1.8972	-4.2815	-1.9431	-4.84	-3.2556	-3.0467	-2.1211	-3.5709
hsa-miR-146a	0.52	-4.5721	1.4763	-4.67	-2.3966	-5.1355	-8.7945	-5.6213
hsa-miR-146b	-2.5847	-6.0846	-2.4375	-5.21	-4.1868	-5.1397	-7.556	-6.1509
hsa-miR-147	14.5346	11.9978	14.7792	11.41	11.4992	12.956	12.3404	12.1321
hsa-miR-148a	-2.3658	-3.9066	-1.6581	-4.4707	-2.6184	-3.0413	-3.306	-2.4225
hsa-miR-148b	0.5762	-2.3867	0.0797	-1.3647	0.585	-1.2324	-3.8653	-1.7391
hsa-miR-149	11.5442	9.0731	9.7944	11.0457	7.7292	8.3006	5.9465	8.2257
hsa-miR-150	9.3183	-3.4775	-0.4952	-6.3485	-2.3151	-5.577	-7.4687	-7.1992
hsa-miR-151	4.8724	4.1054	5.868	0.5567	3.1138	0.0033	-4.2539	-1.009
hsa-miR-152	4.1128	0.0276	4.8428	2.4981	3.5611	0.9525	-2.8183	0.247
hsa-miR-153	14.5442	13.0731	15.5392	11.9002	14.4568	13.928	5.5096	5.4551
hsa-miR-154	14.5442	13.0731	15.5392	11.9002	15.3096	5.1626	4.8502	12.4522
hsa-miR-155	5.8957	0.243	2.9037	-3.0634	-0.2823	-1.0178	-5.0854	-1.8028
hsa-miR-181a	-2.1446	-4.2939	-7.649	-1.3095	-0.6656	-1.0772	-3.1453	-3.3965
hsa-miR-181b	-9.1935	-3.4574	-1.0287	-3.8039	-0.1664	-2.7924	-4.0699	-3.1209
hsa-miR-181c	1.5117	-0.6908	2.0982	1.0707	2.9224	1.2332	-0.2553	-0.5485
hsa-miR-181d	-1.1545	-4.121	-1.1854	-4.475	-1.4398	-1.6978	-4.8709	-3.8162
hsa-miR-182	4.4179	3.5818	3.7049	0.1972	2.8779	2.4311	-2.608	-1.2648

hsa-miR-183	14.5442	11.0431	13.7179	4.7361	7.1455	8.3714	1.7781	3.1266
hsa-miR-184	14.5442	11.5556	15.5392	11.9002	9.983	13.928	11.6474	12.4522
hsa-miR-186	-7.7031	-4.3442	-1.5005	-5.8305	-2.2034	-2.8753	-5.8086	-3.9942
hsa-miR-187	13.3583	8.1004	10.4023	6.8268	12.3863	8.4521	5.0602	5.7977
hsa-miR-188	6.9854	9.006	8.8042	4.9457	5.9801	5.9289	3.7752	3.8387
hsa-miR-190	8.5794	7.1412	8.8575	4.3006	6.7265	5.7658	0.8698	3.1924
hsa-miR-191	-10.5123	-7.1195	-8.5742	-3.2031	-1.1388	-1.1425	-4.2191	-1.2546
hsa-miR-192	9.9178	1.546	6.1504	-1.9777	1.0387	0.8639	-1.6434	-0.4445
hsa-miR-193a	4.0261	2.0883	1.469	1.2754	3.3709	1.2228	1.0588	0.0975
hsa-miR-193b	14.5442	7.0782	10.7566	6.0318	12.7127	13.928	7.6474	5.7152
hsa-miR-194	0.6525	-0.7859	-0.9799	-0.367	-0.4419	0.4649	-17.1376	-1.3399
hsa-miR-195	1.0891	-2.2108	0.0543	-2.9342	-1.8357	-2.9342	-5.277	-3.451
hsa-miR-196a	5.5036	5.1508	6.962	1.5352	3.327	3.8011	0.1341	1.806
hsa-miR-196b	6.1815	4.7048	6.9179	-1.1493	1.0184	2.7794	-1.7228	-0.696
hsa-miR-197	-1.5764	-3.7902	-0.9655	-3.0157	-1.0822	-1.5369	-2.8005	-2.4668
hsa-miR-198	5.4511	8.429	6.2708	4.6458	7.2588	9.3154	9.2475	4.9054
hsa-miR-199a	14.5442	13.0731	10.0157	4.3913	7.68	2.3849	-0.2791	2.2735
hsa-miR-199b	1.4392	-1.6865	0.1885	-0.2891	2.0066	0.6864	0.2043	-0.6868
hsa-miR-200a#	12.4309	13.0731	10.9432	5.4476	9.7896	9.6461	7.5593	11.4889
hsa-miR-200c	2.4966	3.0979	3.7923	0.7708	3.3283	2.02	-0.7517	-0.4433
hsa-miR-202#	13.0288	10.2543	10.1218	11.9002	9.4391	12.6901	6.5836	0.8988
hsa-miR-203	7.7663	13.0731	9.9473	6.1685	9.2215	9.928	4.7828	5.8433
hsa-miR-204	7.9666	8.2111	10.5828	4.9808	9.1218	9.8611	4.4833	6.6988
hsa-miR-205	14.5442	13.0731	15.5392	11.9002	15.3096	13.928	11.6474	12.4522
hsa-miR-206	7.0391	7.1842	7.211	9.2504	6.384	5.937	5.9591	5.1673
hsa-miR-208	12.6765	13.0731	-11.4817	11.9002	11.3096	10.2042	11.6474	12.4522
hsa-miR-210	3.5169	-13.6855	2.7517	2.019	4.9381	2.5967	-1.189	1.4996
hsa-miR-211	14.5442	13.0731	15.5392	7.4358	8.7714	12.2477	11.6474	11.2735
hsa-miR-212	6.1186	13.0731	9.3499	4.153	4.4267	4.5121	3.2512	3.4042
hsa-miR-213	3.0647	1.1968	4.3445	-0.2279	3.4104	1.3179	0.3837	0.6091
hsa-miR-214	4.5575	6.5374	4.51	0.0893	3.9765	7.5102	6.3893	2.097
hsa-miR-215	4.9893	5.2937	4.5572	6.0636	5.519	6.2266	3.5432	3.5706
hsa-miR-216	12.8561	13.0731	11.02	6.196	8.7811	10.1361	11.6474	5.986
hsa-miR-217	14.5442	13.0731	15.5392	11.9002	11.5632	13.928	11.6474	9.9382
hsa-miR-218	5.6518	5.5313	6.5849	3.118	6.626	7.7113	4.8887	4.7201
hsa-miR-219	9.1413	13.0731	10.0457	11.9002	10.431	7.0801	5.1083	8.3831
hsa-miR-220	-20.0281	13.0731	10.8952	11.9002	15.3096	13.928	-7.4916	12.4522
hsa-miR-221	-3.3364	-11.874	-2.9128	-7.109	-2.8123	-4.9636	-8.9432	-5.0644
hsa-miR-222	-3.5418	-6.9756	-3.588	-7.5462	-2.1904	-4.7474	-7.129	-4.893
hsa-miR-223	-13.1209	13.0731	-11.8319	-14.5602	-11.4954	-13.8162	-13.2107	-11.1404
hsa-miR-224	8.083	13.0731	8.3771	4.2982	5.6727	5.1562	4.0026	2.7711
hsa-miR-296	5.7739	6.081	4.0736	2.6409	4.5223	2.4828	-0.294	2.1577
hsa-miR-299-5p	14.5442	13.0731	15.5392	7.9002	15.3096	6.2035	3.8717	8.4522
hsa-miR-301	0.5043	-2.7342	0.2327	-2.6384	0.6695	-1.4006	-4.3073	-1.5424
hsa-miR-302a#	14.5442	13.0731	15.5392	11.9002	15.3096	13.928	11.6474	12.4522
hsa-miR-302b#	12.1715	13.0731	15.339	11.9002	11.9097	13.8375	11.6474	12.4522
hsa-miR-302c#	14.5442	13.0731	15.5392	11.9002	13.5787	13.928	11.315	12.4522
hsa-miR-302d	14.5442	13.0731	-21.0151	11.9002	12.276	13.928	11.6474	12.4522
hsa-miR-320	-2.9748	-2.6584	-2.1556	-5.5046	-2.9122	-3.7731	-5.8105	-5.1274
hsa-miR-323	14.5442	13.0731	12.7731	9.363	12.0733	5.2776	3.4657	7.1641
hsa-miR-324-3p	-14.4613	4.9867	-7.3711	-1.2355	0.3888	1.0009	-3.3167	-1.4419
hsa-miR-324-5p	2.013	1.2813	2.8933	-1.6943	1.6148	1.4343	-2.9799	-0.1441
hsa-miR-325	14.5442	13.0731	15.3598	11.9002	13.3505	13.928	11.6474	12.4522
hsa-miR-326	14.5442	13.0731	9.6641	3.2555	5.4026	2.9147	-0.0639	1.9071
hsa-miR-328	3.7611	2.5434	0.4522	-1.1413	1.6701	-1.2891	-3.009	-0.4162
hsa-miR-330	3.2887	2.886	6.7629	3.0905	5.3474	2.8003	0.971	3.8722

hsa-miR-331	0.9748	-2.2817	0.305	-3.3709	1.3713	-0.5447	-9.4621	-1.9459
hsa-miR-335	1.7909	-0.8351	2.0826	-2.0515	1.5146	0.3686	-3.6843	-1.0939
hsa-miR-337	14.5442	13.0731	15.5392	11.9002	15.3096	8.1354	6.1492	12.4522
hsa-miR-338	1.9907	13.0731	0.6103	0.8067	2.0346	0.9365	-1.0712	0.0772
hsa-miR-339	1.2509	-1.7239	0.8841	-1.0593	2.1095	-0.265	-3.0764	-1.7286
hsa-miR-340	3.0842	1.3051	2.2901	-0.9341	1.5858	0.8611	-2.2665	-0.2347
hsa-miR-342	0.1432	-2.4624	-0.3429	-3.2157	0.5859	-2.7582	-3.1122	-2.972
hsa-miR-345	-9.4532	13.0731	-0.3664	-3.7881	-1.1257	-1.7334	-2.7626	-2.9673
hsa-miR-346	7.5362	13.0731	8.1838	11.9002	5.1184	5.742	6.3044	5.4274
hsa-miR-361	-0.8233	-13.2668	-9.2543	-1.8642	-0.2093	-1.1311	-2.7194	-1.6524
hsa-miR-365	1.445	-1.0411	0.0553	-3.4772	-0.1576	-1.0269	-0.3419	-0.0149
hsa-miR-367	14.5442	13.0731	15.5392	11.9002	-15.7351	13.928	11.6474	12.4522
hsa-miR-368	14.5442	13.0731	15.5392	11.9002	15.3096	7.422	6.3231	12.4522
hsa-miR-369-3p	14.5442	13.0731	15.5392	11.9002	15.3096	6.6944	3.4091	7.0666
hsa-miR-369-5p	14.5442	13.0731	15.5392	7.2149	15.3096	5.6273	5.0333	12.4522
hsa-miR-370	14.5442	4.7251	6.5392	9.4959	5.4517	2.6521	2.3527	2.6695
hsa-miR-371	13.3321	13.0731	13.3792	11.9002	11.11	13.4132	9.668	12.4522
hsa-miR-372	12.8434	13.0731	15.5392	11.9002	15.3096	13.928	11.6474	12.4522
hsa-miR-373#	14.5442	13.0731	12.8776	11.9002	7.5092	12.4631	11.6474	12.4522
hsa-miR-374	-1.7193	-3.798	-1.565	-4.7459	-2.5729	-3.4194	-6.1834	-3.9183
hsa-miR-375	6.9281	13.0731	9.3535	8.9258	7.1963	8.3772	6.7436	4.8426
hsa-miR-376a	6.5034	8.3044	8.0398	6.1565	6.4424	2.1653	0.0272	4.7366
hsa-miR-378	-12.0311	2.451	3.5731	-1.632	1.9535	0.5495	-0.241	-0.9755
hsa-miR-379	12.1762	13.7639	15.4059	5.9253	11.0784	3.3864	2.7589	5.5814
hsa-miR-380-3p	13.8948	13.7639	15.4059	11.8328	11.3384	9.9238	8.5597	7.1951
hsa-miR-381	5.0106	13.7639	11.9699	4.1662	8.5515	-20.2104	5.8244	5.0142
hsa-miR-382	13.6257	13.7639	9.1835	3.3135	10.9774	2.2278	0.9874	2.3445
hsa-miR-383	6.9914	9.2017	9.3348	5.9829	8.2871	7.8097	5.6537	5.4966
hsa-miR-409-5p	13.8948	-20.8606	15.4059	11.8328	15.5098	6.2044	4.6987	10.8642
hsa-miR-422a	-13.3543	4.1892	5.7727	0.893	6.1987	4.1188	3.9655	1.4732
hsa-miR-422b	3.591	10.4239	11.3589	-1.7087	0.7526	-0.6496	-1.0343	-2.8076
hsa-miR-423	-0.7577	13.7639	3.5302	-5.6133	-0.9733	-0.704	-3.3943	-4.5821
hsa-miR-424	-0.6685	-0.4269	15.4059	-4.0839	-0.193	-0.3676	-1.6953	-4.4599
hsa-miR-425	-0.6497	-0.8073	0.7539	-11.5792	-0.0624	-1.3024	-2.6237	-3.3679
hsa-miR-429	5.7816	5.61	6.4017	3.4765	10.0526	4.3472	3.364	1.6309
hsa-miR-432	-0.3409	11.7721	6.1537	10.3946	10.1462	1.0336	0.9916	4.0029
hsa-miR-433	8.5233	7.001	7.5263	4.1146	5.9726	3.8218	3.4203	3.5084
hsa-miR-449	10.448	11.7319	8.6597	4.6424	8.5582	8.154	3.1567	4.559
hsa-miR-450	2.6208	2.9084	3.3918	-0.0275	4.8302	1.9161	0.8692	-0.0668
hsa-miR-451	-3.9844	-5.8154	-3.2726	-14.0495	-10.1588	-3.7485	-10.4376	-14.4115
hsa-miR-452	4.4884	7.9955	6.8941	2.4911	5.4477	8.825	8.0156	3.1597
hsa-miR-485-5p	8.4511	8.0408	7.6478	3.872	6.1683	5.6412	4.4299	4.4731
hsa-miR-489	13.6244	13.7639	9.9635	4.9616	15.787	10.8463	12.6281	10.8642
hsa-miR-490	9.5189	5.6944	8.3019	7.1747	6.9545	4.3671	-0.2973	0.7704
hsa-miR-491	2.51	1.2975	2.0027	-3.5007	4.0275	0.1127	1.358	-1.6809
hsa-miR-494	8.2609	10.3186	6.6304	4.5942	4.5894	3.8762	3.7144	4.4236
hsa-miR-496	13.8948	13.7639	11.2502	8.7353	12.1133	5.9854	4.2158	5.8072
hsa-miR-497	3.6352	6.7905	4.9789	1.4443	5.9012	3.1164	-2.1747	0.1442
hsa-miR-500	4.6811	8.4598	-18.024	1.7606	5.5184	3.0037	3.7777	1.4266
hsa-miR-501	4.3366	5.7213	5.9865	-0.0746	4.4736	2.6986	1.7492	0.9714
hsa-miR-502	5.8496	10.1176	11.7265	2.3709	5.8237	3.56	3.8184	3.1328
hsa-miR-505	-0.2722	13.7639	15.4059	-2.5153	1.5826	-0.7421	-2.0161	-3.2189
hsa-miR-506	13.8948	13.7639	15.4059	10.5201	10.8834	12.2073	7.4919	10.8642
hsa-miR-508	13.8948	13.7639	15.4059	4.7883	9.2934	13.8023	6.3129	3.9973
hsa-miR-509	10.9874	13.0466	9.3647	5.8754	7.4296	7.921	6.2981	4.7996
hsa-miR-510	10.7898	11.2268	11.5965	8.9684	10.2142	11.6737	12.1289	10.8642

hsa-miR-511	8.4207	13.7639	8.8702	4.4744	8.3376	9.0623	12.6281	10.8642
hsa-miR-512-5p	10.9253	13.7639	14.0855	7.8107	9.6577	9.1636	10.596	10.0045
hsa-miR-513	13.8948	13.7639	15.4059	11.8328	11.9976	13.8023	12.6281	10.8642
hsa-miR-514	11.4358	13.7639	10.6326	6.9426	10.9036	9.086	5.9554	3.5564
hsa-miR-515-3p	11.2334	11.3662	10.2692	8.7228	8.2152	9.6969	8.0401	9.0659
hsa-miR-515-5p	13.8948	13.7639	-21.1384	-11.3685	15.787	13.8023	11.6651	10.8642
hsa-miR-516-3p	5.4259	5.5799	7.158	3.5903	7.9903	6.3718	6.5802	3.0712
hsa-miR-517a	13.8948	13.7639	-20.0104	-17.6634	12.7143	13.2993	12.6281	10.8642
hsa-miR-517b	13.8948	13.7639	13.255	11.8328	12.4408	13.8023	12.6281	10.8642
hsa-miR-517c	13.8948	13.7639	15.4059	6.8863	10.9991	13.8023	9.6853	10.8642
hsa-miR-518a	8.4994	13.7639	14.0581	8.3001	9.827	8.8025	8.3952	7.1447
hsa-miR-518b	4.2038	5.4889	5.581	1.0821	4.7752	6.5236	7.6729	1.6836
hsa-miR-518c	11.8678	11.1472	15.4059	9.9809	11.4779	11.61	11.3622	8.7229
hsa-miR-518d	8.9405	13.7639	15.0175	7.5822	8.4317	9.8782	7.7075	6.8455
hsa-miR-518e	13.8101	11.5727	11.7521	10.2599	8.9221	7.9072	8.6281	7.9346
hsa-miR-519b	13.8948	13.7639	15.4059	11.8328	15.787	13.8023	12.6281	10.8642
hsa-miR-519c	9.0418	13.7639	14.6571	9.6598	12.1378	10.3403	11.4539	8.2154
hsa-miR-519d	9.9983	11.6882	12.483	6.5422	7.6534	8.7271	7.401	7.0675
hsa-miR-519e	8.6473	13.7639	9.5963	8.5727	7.8408	7.5929	10.4548	-13.8244
hsa-miR-520a	13.8948	13.7639	13.4149	7.1854	8.5836	10.1683	11.5044	6.8642
hsa-miR-520b	13.8948	13.7639	12.9271	8.9097	6.4162	11.0214	10.4481	7.8847
hsa-miR-520c	13.8948	13.7639	12.5931	8.5487	12.4226	10.9877	12.6281	8.9787
hsa-miR-520d	13.8948	13.7639	7.3446	8.1771	10.2002	13.0151	11.8095	10.8642
hsa-miR-520e	13.8948	-9.5494	15.4059	-15.2768	15.787	13.8023	12.6281	10.8642
hsa-miR-520f	-10.9124	13.7639	15.4059	7.8328	11.0708	7.8172	12.449	10.037
hsa-miR-520g	13.8948	-10.3076	-14.5941	11.8328	13.222	13.8023	12.6281	0.5475
hsa-miR-520h	13.8948	13.7639	15.4059	10.811	9.8531	9.8023	12.6281	10.8642
hsa-miR-521	13.8948	13.7639	15.4059	11.8328	13.3526	13.7002	12.6281	10.8642
hsa-miR-522	13.8948	-14.2361	15.4059	11.8328	11.787	13.8023	11.1914	10.8642
hsa-miR-523	-15.1052	-14.5719	14.0911	11.8328	15.1398	13.8023	12.6281	10.8642
hsa-miR-526a	13.8948	13.7639	15.4059	8.4996	11.6769	13.8023	-7.579	7.4828
hsa-miR-526b	-11.6906	13.7639	15.4059	-9.781	14.8718	13.16	12.6281	10.8642
